# Impact of Health Insurance Status on HbA1c Control Among United States Adults With Type 2 Diabetes: A Cross-Sectional Analysis of Nationally Representative Data

**DOI:** 10.7759/cureus.91421

**Published:** 2025-09-01

**Authors:** Basit O Bolarinwa, Chiamaka J Ohiri, Rasheedat A Sadiq-Onilenla, Sunday P Iziogo, Okelue E Okobi

**Affiliations:** 1 Internal Medicine, Richmond Gabriel University, Kingstown, VCT; 2 Neurology, University of Pécs, Pécs, HUN; 3 Health Sciences, Franklin University, Columbus, USA; 4 Emergency, Northwest Hospital, Randallstown, USA; 5 Family Medicine, Larkin Community Hospital Palm Springs Campus, Miami, USA; 6 Family Medicine, IMG Research Academy and Consulting, Homestead, USA

**Keywords:** glycemic control, hba1c, health disparities, health insurance, nhanes, type 2 diabetes

## Abstract

Background: Glycemic control is essential for reducing complications in individuals with type 2 diabetes. Health insurance is a known determinant of access to care, yet its relationship with glycemic outcomes remains underexplored in nationally representative data. Therefore, the objective of this study is to assess the association between health insurance status and glycemic control among United States (US) adults with diagnosed type 2 diabetes (diagnostic A1c cutoff ≥ 6.5%) using National Health and Nutrition Examination Survey (NHANES) data. We hypothesized that uninsured adults with type 2 diabetes would have higher odds of poor glycemic control.

Methods: A cross-sectional analysis was conducted using data from the NHANES 2013-2018 cycles. Adults aged ≥18 years with diagnosed type 2 diabetes were included. Survey-weighted logistic regression models examined the association between insurance status and poor glycemic control (glycated hemoglobin (HbA1c) ≥ 7%), adjusting for demographic, clinical, and behavioral covariates. Descriptive and inferential statistics accounted for the complex survey design.

Results: Among 229 million weighted US adults with diagnosed type 2 diabetes, 195,397,912 (85%) were insured. Among those with poor glycemic control, 18,684,942 (87%) were insured and 2,901,970 (13%) were uninsured. Insurance status was not significantly associated with glycemic control in either the demographically adjusted model (odds ratio (OR) = 1.15, 95% confidence interval (CI): 0.91-1.45) or the fully adjusted model (OR = 1.12, 95% CI: 0.89-1.41). Significant predictors of poor glycemic control included age, gender, body mass index (BMI), income, and race/ethnicity.

Conclusion: Health insurance alone may not ensure optimal glycemic control. Addressing broader social and clinical factors is essential to improving outcomes in diabetes care.

## Introduction

Type 2 diabetes mellitus (T2DM) is a metabolic condition of a chronic nature, afflicting millions of people in the United States (US) and placing a huge burden on both the state of health of the population and the healthcare system [1]. The Centers for Disease Control and Prevention (CDC) states that more than 38 million Americans have diabetes, and an estimated 90%-95% of cases are type 2 diabetes [2]. Insulin resistance and reduced insulin secretion are pathophysiological features of T2DM, which lead to chronic hyperglycemia and, over time, may result in severe complications such as cardiovascular disease, nephropathy, retinopathy, neuropathy, and limb amputation [3-5]. Achievement of glycemic control, operationalized as glycated hemoglobin (HbA1c), with poor control defined as HbA1c ≥ 7%, is a fundamental clinical objective in the treatment of diabetes because it reflects historical average blood glucose levels over the preceding 2-3 months and is closely associated with long-term health outcomes [6].

The American Diabetes Association (ADA) recommended that most non-pregnant adults should have an HbA1c of below 7% (A1c ≥ 6.5%) to minimize the risk of complications [7]. Given the number of patients with inadequate glycemic control, few can reach and sustain ideal glycemic control, especially those who are underserved because of socioeconomic factors [8,9]. Health insurance status has been established as a critical structural factor among other determinants that contribute to glycemic outcomes [10]. The availability of insurance coverage and low-cost, sustained healthcare is a critical factor in determining the capacity of people to obtain early diagnosis, further access to biologic therapies (e.g., insulin) and oral hypoglycemic agents, and attend follow-up visits and obtain regular measurements of HbA1c [11,12]. Uninsured persons could face care delays, reduced treatment compliance, and minimal utilization of preventive healthcare, which can be the key factors of impaired glycemic control and increase the disease burden [13].

In the United States, access to healthcare is a basic gateway that requires health insurance [14]. Although attempts to improve insurance coverage have been successfully implemented with the help of the Affordable Care Act (ACA), there is still a gap, especially in communities with a low-income rate and racial or ethnic minorities, and those belonging to the young adult community [15,16]. It has been found that insurance coverage increases the tendency of people to use healthcare facilities, follow prescribed medical procedures, and have greater control over their chronic conditions [17]. However, there are more findings regarding the connection between insurance status and glycemic control in the representatives of type 2 diabetes.

The role of insurance in the process of glycemic outcomes is especially relevant to be understood since disease burden increases, and diabetes is a condition that negatively impacts vulnerable populations [18]. HbA1c can be a measure of individual disease management as well as an indicator of the health of the population and a measure of the quality of access to the health system [19].

Data collected by a nationally representative source in the United States, the National Health and Nutrition Examination Survey (NHANES), provides good insights into whether differences in HbA1c control disparities occur between insured and uninsured adults [20]. This study aims to test the hypothesis that insured US adults with type 2 diabetes have better glycemic control (lower odds of HbA1c ≥ 7%) compared to uninsured adults, after adjusting for demographic, socioeconomic, and clinical factors.

## Materials and methods

Study design and data sources

This study utilized a cross-sectional design based on data from the NHANES, a nationally representative survey conducted by the National Center for Health Statistics (NCHS) [21]. NHANES collects detailed health-related data through household interviews, physical examinations, and laboratory tests using a complex, multistage probability sampling method. The analysis included data from 2013-2018 cycles that provided relevant information on diabetes status, HbA1c measurements, and health insurance coverage. The survey’s design enables generalization of findings to the non-institutionalized adult US population.

Study population

The study sample comprised adults aged 18 years and older who reported a diagnosis of T2DM. T2DM was identified through self-reported physician diagnosis, excluding those likely to have type 1 diabetes, defined as a diagnosis before age 30 and current use of insulin without any oral hypoglycemic agents. Participants were included only if they had complete data for all variables used in the regression models. Individuals with missing information on any key variable were excluded using complete-case analysis to ensure the accuracy and integrity of multivariable estimations.

Variables

The primary exposure variable was health insurance status, categorized as either “insured” or “uninsured” based on participant responses at the time of interview. The primary outcome was glycemic control, measured by hemoglobin A1c (HbA1c) levels, obtained from laboratory data. Poor glycemic control was defined as HbA1c ≥ 7%, consistent with the American Diabetes Association Standards of Care (2024) [7]. Covariates included demographic and clinical factors such as age, sex, race/ethnicity, education level, annual household income, body mass index (BMI), smoking status, and duration of diabetes, all selected based on their known association with glycemic outcomes and access to care. Smoking status was coded as never, former, or current smoker: never = reported having smoked fewer than 100 cigarettes in their lifetime; former = reported having smoked at least 100 cigarettes in their lifetime, but not currently smoking; current = reported having smoked at least 100 cigarettes in their lifetime and currently smoking (every day or some days). This classification follows standard NHANES/NCHS practice.

Statistical analysis

All statistical analyses were performed using STATA version 18 (StataCorp LLC, College Station, TX), incorporating survey weights, strata, and primary sampling units provided by NHANES to account for the complex multistage sampling design. These adjustments ensure nationally representative estimates and valid standard errors. Descriptive statistics were used to summarize the characteristics of the study population according to health insurance status. Continuous variables were presented as means with standard deviations (SD) and compared using t-tests. For categorical variables, group differences were assessed using design-adjusted F-statistics derived from Rao-Scott chi-square tests, which are appropriate for complex survey data and account for weighting and clustering.

To examine the association between health insurance status and poor glycemic control (defined as HbA1c ≥ 7%), survey-weighted logistic regression models were fitted using the svy commands in STATA. These models accounted for the stratified, clustered nature of the NHANES sample and incorporated full sampling weights to yield valid population-level inferences. Multivariable logistic regression analyses were conducted in sequential steps, beginning with unadjusted models and progressing to models adjusted for sociodemographic and clinical covariates, including age, sex, race/ethnicity, education, income, BMI, smoking status, and duration of diabetes.

A complete-case (listwise deletion) strategy was applied to handle missing data. Participants with missing information on any variable included in the regression models were excluded from the analysis. Although multiple imputation is commonly used to address missingness, it was not implemented in this study due to the methodological complexity involved in applying imputation procedures that appropriately reflect NHANES’s multistage sampling structure. Specifically, most standard imputation methods are not designed to accommodate survey weights, stratification, and clustering simultaneously, making their integration statistically impractical in this context.

Ethical considerations

The analysis was based on publicly available, de-identified NHANES data and therefore did not require institutional review board (IRB) approval. NHANES survey protocols are reviewed and approved by the NCHS Research Ethics Review Board, and all participants provide informed consent before data collection.

## Results

Table 1 presents the weighted characteristics of US adults with type 2 diabetes, stratified by health insurance status.

**Table 1:** Characteristics of US Adults With Type 2 Diabetes by Health Insurance Status (NHANES 2013-2018) Survey-weighted estimates Continuous variables are presented as mean ± SD and were compared using independent t-tests. Categorical variables are presented as counts (number) and weighted percentages (%) and compared using design-adjusted F-statistics. Most variables were derived from NHANES data based on recoding or categorization (health insurance status, income level, smoking status, education level, and glycemic control thresholds). Significance level: p < 0.05, p < 0.01, p < 0.001 -: intentionally left blank, NHANES: National Health and Nutrition Examination Survey, SD: standard deviation, HbA1c: glycated hemoglobin

Variable	Insured (n = 195,397,912)	Uninsured (n = 34,572,133)	T/F-test	p-value
Age (years, mean ± SD)	49.41 ± 16.95	39.01 ± 14.61	t = -25.43	<0.001
Body mass index (kg/m^2^, mean ± SD)	29.46 ± 6.98	29.45 ± 8.02	t = -0.06	0.95
HbA1c ≥ 7% (number (%))	-	-	F = 2.16	0.15
Good control	176,712,970 (85%)	31,670,163 (15%)	-	-
Poor control	18,684,942 (87%)	2,901,970 (13%)	-	-
Gender (number (%))	-	-	F = 84.35	<0.001
Male	91,107,892 (83%)	19,254,100 (17%)	-	-
Female	104,290,020 (87%)	15,318,033 (13%)	-	-
Smoking status (number (%))	-	-	F = 66.01	<0.001
Never	113,597,612 (87%)	17,202,526 (13%)	-	-
Former	50,052,699 (89%)	6,439,807 (11%)	-	-
Current	31,747,600 (74%)	10,929,800 (26%)	-	-
Education level (number (%))	-	-	F = 173.46	<0.001
Less than high school	21,162,485 (67%)	10,233,303 (33%)	-	-
High school grad	42,787,565 (80%)	10,695,242 (20%)	-	-
Some college+	131,447,862 (91%)	13,643,588 (9%)		
Income level (number (%))	-	-	F = 207.07	<0.001
Below poverty	20,023,743 (67%)	10,002,275 (33%)	-	-
Low-mid income	61,495,085 (80%)	15,073,854 (20%)	-	-
High income	113,879,083 (92%)	9,496,004 (8%)	-	-
Race/ethnicity (number (%))	-	-	F = 1.7.25	<0.001
Mexican American	12,508,078 (61%)	7,918,455 (39%)	-	-
Other Hispanic	10,531,804 (72%)	4,061,502 (28%)	-	-
Non-Hispanic White	132,596,822 (90%)	14,553,861 (10%)	-	-
Non-Hispanic Black	20,868,733 (80%)	5,300,503 (20%)	-	-
Non-Hispanic Asian	18,892,473 (87%)	2,737,811 (13%)	-	-

Compared to uninsured adults, those with health insurance were significantly older (49.41 ± 16.95 versus 39.01 ± 14.61 years, p < 0.001). No meaningful difference was observed in average BMI between insured and uninsured individuals (29.46 versus 29.45 kg/m², p = 0.95).

Glycemic control, based on HbA1c levels, was more prevalent among insured adults, with 176,712,970 (85%) achieving good control compared to 31,670,163 (15%) among the uninsured. Poor glycemic control was slightly higher in the insured group (18,684,942 (87%) versus 2,901,970 (13%)), although this difference was not statistically significant (p = 0.15).

Gender distribution differed significantly by insurance status, with a higher proportion of insured women (104,290,020 (87%)) than uninsured (15,318,033 (13%)) (p < 0.001). Smoking status was also significantly associated with insurance status. Among the uninsured, 10,929,800 (26%) were current smokers, compared to 31,747,600 (74%) current smokers who were insured (F = 66.01, p < 0.001).

Socioeconomic factors revealed striking disparities. Adults with lower education and income levels were disproportionately uninsured. For instance, only 21,162,485 (67%) adults with less than a high school education were insured compared to 131,447,862 (91%) of those with some college education or higher (p < 0.001). Similarly, 10,002,275 (33%) individuals below the poverty line were uninsured, compared to just 9,496,004 (8%) in the high-income group.

Racial and ethnic disparities were evident. Uninsurance rates were highest among Mexican Americans (7,918,455 (39%)) and other Hispanics (4,061,502 (28%)), compared to 14,553,861 (10%) among non-Hispanic Whites (p < 0.001).

Table 2 displays the results from two survey-weighted multivariable logistic regression models assessing the relationship between health insurance status and glycemic control among US adults with diagnosed type 2 diabetes. Poor glycemic control was again defined as having an HbA1c level ≥ 7%. The first model adjusts for demographic variables (age, sex, race/ethnicity, education, and income), while the second model extends the adjustment to include clinical and behavioral factors, specifically body mass index (BMI) and smoking status.

**Table 2:** Association Between Health Insurance Status and Poor Glycemic Control (HbA1c ≥ 7%) Among US Adults With Type 2 Diabetes OR and 95% confidence intervals in brackets Significance level: *p < 0.05, **p < 0.01, ***p < 0.001 -: intentionally left blank, OR: odds ratio, HbA1c: glycated hemoglobin

HbA1c ≥ 7% (0 = good, 1 = poor)	Demographically adjusted model (n = 15,998)	Fully adjusted model (n = 15,998)
Health insurance status	-	-
Insured	1.148 (0.911,1.447)	1.119 (0.890,1.407)
Age in years at screening	1.025 (1.021,1.030)^ ***^	1.026 (1.021,1.031)^***^
Gender	-	-
Female	0.729 (0.619,0.859)^***^	0.693 (0.593,0.810)^***^
Education level	-	-
High school Grad	0.927 (0.780,1.101)	0.875 (0.735,1.042)
Some college+	0.822 (0.683,0.989)^*^	0.791 (0.660,0.949)^*^
Income level	-	-
Low-mid income	0.915 (0.746,1.122)	0.915 (0.743,1.127)
High income	0.766 (0.611,0.960)^*^	0.792 (0.624,1.006)
Race/ethnicity	-	-
Other Hispanic	0.901 (0.694,1.170)	0.972 (0.753,1.257)
Non-Hispanic White	0.608 (0.473,0.782)^***^	0.648 (0.506,0.831)^**^
Non-Hispanic Black	1.328 (1.085,1.624)^**^	1.341 (1.091,1.648)^**^
Non-Hispanic Asian	1.038 (0.826,1.304)	1.252 (0.998,1.569)
Body mass index (kg/m²)	-	1.051 (1.039,1.063)^***^
Smoking status	-	-
Former	-	1.056 (0.901,1.237)
Current	-	0.990 (0.783,1.252)

Across both models, health insurance status was not significantly associated with poor glycemic control. In the demographically adjusted model, insured individuals had 15% higher odds of poor HbA1c control compared to uninsured adults (OR: 1.15, 95% CI: 0.91-1.45, p > 0.05), although this result was not statistically significant. After further adjustment for BMI and smoking behavior in the full model, the association remained non-significant (OR: 1.12, 95% CI: 0.89-1.41, p > 0.05). These findings suggest that, within this nationally representative sample, health insurance coverage alone may not be a determining factor in achieving optimal glycemic control among adults with type 2 diabetes. In contrast, several sociodemographic and clinical covariates were significantly associated with glycemic outcomes. Age at screening was positively associated with poor glycemic control in both models. Each one-year increase in age was associated with 2.6% higher odds of poor control in the fully adjusted model (OR: 1.03, 95% CI: 1.02-1.03, p < 0.001), indicating that glycemic management becomes more challenging with advancing age.

Sex differences in glycemic control were also observed. Compared to men, women had significantly lower odds of poor glycemic control across both models. In the fully adjusted model, women had 31% reduced odds of poor control (OR: 0.69, 95% CI: 0.59-0.81, p < 0.001).

Educational attainment showed a protective effect, particularly for individuals with some college education or higher. Compared to adults with less than a high school education, those with some college had 21% lower odds of poor glycemic control (OR: 0.79, 95% CI: 0.66-0.95, p < 0.05) in the fully adjusted model. While high school graduates also exhibited reduced odds, this association did not reach statistical significance.

Income levels in glycemic control were also evident. Participants in the highest income group had 23% lower odds of poor control compared to those below the federal poverty level in the demographically adjusted model (OR: 0.77, 95% CI: 0.61-0.96, p < 0.05), although this association attenuated and lost significance in the fully adjusted model (OR: 0.79, 95% CI: 0.62-1.01).

Race/ethnicity emerged as a significant predictor in both models. Compared to Mexican Americans, non-Hispanic White adults had significantly lower odds of poor glycemic control (OR: 0.65, 95% CI: 0.51-0.83, p < 0.01), while non-Hispanic Black individuals had elevated odds (OR: 1.34, 95% CI: 1.09-1.65, p < 0.01).

Clinical characteristics were also associated with glycemic control. In the fully adjusted model, each one-unit increase in BMI was associated with a 5% increase in the odds of poor glycemic control (OR: 1.05, 95% CI: 1.04-1.06, p < 0.001), highlighting obesity as a key clinical factor influencing diabetes outcomes.

Finally, smoking status did not show significant associations with glycemic control. Neither former nor current smokers differed meaningfully from never smokers in terms of HbA1c outcomes, after adjusting for other covariates.

## Discussion

Using the NHANES 2013-2018 data, this study sought to investigate whether health insurance status is independently associated with glycemic control among US adults with type 2 diabetes. Contrary to our initial hypothesis, health insurance coverage was not significantly linked to poor glycemic control (HbA1c ≥ 7%) in either the demographically adjusted model (OR: 1.15, 95% CI: 0.91-1.45) or the fully adjusted model (OR: 1.12, 95% CI: 0.89-1.41). These results imply that, while insurance is necessary for access, it may be insufficient by itself to produce better glycemic outcomes; other sociodemographic and clinical determinants appear to be more influential.

Age emerged as a consistent risk factor; each additional year was associated with approximately 3% increased odds of poor glycemic control (OR: 1.03, 95% CI: 1.02-1.03, p < 0.001), reflecting known age-related metabolic decline and complexity in managing multiple comorbidities. This pattern aligns with epidemiologic evidence, such as findings from the Australian National Diabetes Audit, which reported that older adults with type 2 diabetes exhibit significantly higher odds of having HbA1c above target compared to younger patients [22].

Female sex was significantly protective, with women exhibiting approximately 31% lower odds of poor glycemic control in the full model (OR: 0.69, 95% CI: 0.59-0.81, p < 0.001), suggesting potential behavioral or physiological differences in diabetes management and/or disease progression by sex. This observation aligns with prior research demonstrating that women engage more frequently in diabetes self-care behaviors such as monitoring, dietary management, and symptom recognition, which may underlie their comparatively better glycemic control [23].

Socioeconomic indicators, including education and income levels, demonstrated meaningful associations with HbA1c control. Adults with some college education had notably lower odds of poor control (OR: 0.79, 95% CI: 0.66-0.95, p < 0.05), and those in the highest income bracket experienced similarly reduced odds in the demographic model (OR: 0.77, 95% CI: 0.61-0.96, p < 0.05). These patterns suggest socioeconomic disparities in glycemic outcomes, possibly reflecting differences in access to resources, dietary patterns, or diabetes self-management education. The findings reinforce the role of higher education and financial resources in facilitating access to diabetes knowledge, self-management tools, and healthcare access factors documented in prior national analyses.

Racial and ethnic disparities in glycemic control were evident. Non-Hispanic Black adults had higher odds of poor control (OR: 1.34, 95% CI: 1.09-1.65, p < 0.01), while non-Hispanic White individuals had significantly lower odds (OR: 0.65, 95% CI: 0.51-0.83, p < 0.01), compared to Mexican Americans. These findings underscore the persistent racial and ethnic disparities in diabetes management and may reflect systemic differences in care quality, cultural barriers, or comorbid disease burden. These disparities align with recent findings that racial inequities in diabetes outcomes persist even among insured adults, indicating that insurance status alone does not eliminate systemic barriers to quality care.

From a clinical standpoint, higher BMI was consistently associated with poorer glycemic control (OR: 1.05 per unit increase, 95% CI: 1.04-1.06, p < 0.001), reinforcing the well-established link between obesity and insulin resistance. Mechanistically, obesity-associated adipose tissue inflammation and related inflammatory signaling pathways have been implicated in the development of systemic insulin resistance and impaired glucose homeostasis [24,25]. It has consistently been observed in several studies to be one of the major reported factors affecting glycemic control, as also observed in a recent study done in Ethiopia, on diabetic patients, which showed that 68% had poor glycemic control associated with high low-density lipoprotein (LDL) cholesterol, and several other factors [9]. A study involving the addition of tirzepatide to the insulin treatment regimen showed a statistically significant improvement in both glycemic control and body weight after 40 days [8]. This was observed in the fully adjusted model as a positive association between high BMI and poor glycemic control. It further highlights the importance of weight management in the management of diabetes.

Interestingly, smoking status did not significantly influence glycemic control in our fully adjusted model. While smoking is often connected to cardiovascular risks, its direct impact on HbA1c is less clear and may be moderated by lifestyle or treatment factors not captured in our dataset.

Further, the findings of this study suggest that while insurance is a necessary foundation for healthcare access, it is not sufficient to guarantee effective diabetes control. Achieving HbA1c targets likely requires the integration of services such as diabetes education, medication management, obesity interventions, and culturally tailored care, especially for racial minority groups. This aligns with broader value-based and patient-centered strategies, which advocate for enhanced coverage of non-clinical services such as education and weight loss interventions.

This study has utilized the NHANES 2013-2018 data to establish whether the insurance status correlates with poor glycemic control (HbA1c ≥ 7%). Despite the observed sociodemographic disparities in having health insurance, we establish that being insured or not had little to no major impact on glycemic control among US adults with type 2 diabetes. Rather, age, sex, BMI, income, and race/ethnicity all proved to be predictors of glycemic control, indicating that coverage is not the complete picture of diabetes management. Similarly, the practical obstacles, including high costs of out-of-pocket, insufficient insurance coverage on diabetes self-management education and supplies, affordable medicines and adherence, and fragmentation of care, or even social determinants of health, may be blunting the effect of having nominal insurance on HbA1c outcomes. Thus, this article suggests that the key objectives of policy and clinical initiatives include not only expanding coverage but also increasing affordability, continuity, comprehensiveness of diabetes-related benefits, and social supports to make a significant change associated with glycemic control.

Strengths and limitations

We note several strengths of this study. First, the analysis used a large, nationally representative sample (NHANES 2013-2018) with survey weights that permit population-level inferences for non-institutionalized US adults. Second, HbA1c was measured in standardized clinical laboratories using validated methods, avoiding reliance on self-report for the primary outcome. Third, our analytic approach incorporated survey-weighted regression methods that account for NHANES’ complex multistage sampling, and we adjusted for a pre-specified set of sociodemographic and clinical covariates to reduce confounding. Finally, the large effective sample size provided adequate precision to estimate associations across key subgroups, increasing the generalizability of our findings to US adults with diagnosed type 2 diabetes.

This study has several limitations that warrant careful consideration. First, the cross-sectional design precludes causal inference; observed associations may reflect reverse causation or unmeasured confounding. Second, we used a complete-case (listwise deletion) approach to handle missing data. If missingness was not completely at random, this could introduce selection bias and distort effect estimates; we therefore note that the analytic sample may differ from the full NHANES sample on unobserved characteristics. Third, the measure of “insurance status” is intentionally simple (insured versus uninsured) and does not capture important dimensions of coverage such as plan type (e.g., Medicaid, Medicare, employer-sponsored, and marketplace), benefit generosity, cost-sharing (deductibles/copays), prescription drug coverage, or continuity of coverage over time. These factors could materially influence access to medications, diabetes supplies, and services (e.g., diabetes self-management education), and their omission may attenuate or obscure true relationships between coverage and glycemic control. Fourth, several clinically important variables were unavailable or had high missingness in the public dataset (e.g., duration of diabetes was >85% missing in our sample), limiting our ability to adjust for disease chronicity. Fifth, key mediators such as medication adherence, healthcare utilization frequency, dietary patterns, and psychosocial factors were not measured or were imperfectly captured, raising the possibility of residual confounding. Finally, ascertainment of diagnosed diabetes relied on self-report of a prior physician diagnosis (as in many population surveys), which may be affected by recall bias. Future work should consider richer insurance metrics (including plan type and continuity), longitudinal designs, sensitivity analyses stratified by insurance type, and missing data approaches that incorporate survey design (e.g., multiple imputation methods that accommodate complex survey weights or inverse probability weighting) to assess the robustness of findings.

## Conclusions

In this nationally representative analysis of US adults with type 2 diabetes, health insurance status was not significantly associated with glycemic control after adjusting for demographic, clinical, and behavioral factors. Instead, younger age, female gender, higher education, and higher income were independently associated with better HbA1c outcomes, while non-Hispanic White, non-Hispanic Black, and higher BMI predicted poorer control. These findings suggest that while insurance coverage is essential, improving glycemic outcomes may require addressing broader social, behavioral, and clinical determinants of health. However, the absence of a statistically significant association between insurance status and glycemic control may reflect measurement limitations (binary insurance measure and lack of plan detail), residual confounding, and the cross-sectional design, rather than definitive evidence that no relationship exists. Future research should explore the role of insurance quality, access to care, and diabetes self-management resources to better inform policies aimed at reducing disparities in diabetes control. In particular, longitudinal study designs, richer insurance metrics (e.g., plan type, benefit generosity, cost-sharing, and continuity of coverage), and mediation analyses are recommended to more precisely probe causal pathways and clarify how different dimensions of coverage affect glycemic outcomes.
